# The HIV Care Continuum among Female Sex Workers: A Key Population in Lilongwe, Malawi

**DOI:** 10.1371/journal.pone.0147662

**Published:** 2016-01-25

**Authors:** Kathryn Elizabeth Lancaster, Kimberly A. Powers, Thandie Lungu, Pearson Mmodzi, Mina C. Hosseinipour, Katy Chadwick, Vivian F. Go, Brian W. Pence, Irving F. Hoffman, William C. Miller

**Affiliations:** 1 Department of Epidemiology, Gillings School of Global Public Health, The University of North Carolina at Chapel Hill, Chapel Hill, North Carolina, United States of America; 2 UNC Project Malawi, University of North Carolina at Chapel Hill, Lilongwe, Malawi; 3 Division of Infectious Diseases, School of Medicine, University of North Carolina at Chapel Hill, Chapel Hill, North Carolina, United States of America; 4 Theatre for a Change, Lilongwe, Malawi; 5 Department of Health Behavior, Gillings School of Global Public Health, The University of North Carolina at Chapel Hill, Chapel Hill, North Carolina, United States of America; University of Toronto Dalla Lana School of Public Health, CANADA

## Abstract

**Objective:**

The HIV care continuum among female sex workers (FSW), a key population, has not been well characterized, especially within the generalized epidemics of sub-Saharan Africa. This was the first study to characterize the HIV care continuum among FSW in Lilongwe, Malawi.

**Methods:**

From July through September 2014, we used venue-based sampling to enroll 200 adult FSW in Lilongwe, Malawi into a cross-sectional evaluation assessing HIV care continuum outcomes. Seropositive FSW, identified using HIV rapid testing, received rapid CD4 counts in addition to viral loads using dried blood spots. We calculated proportions of HIV-infected FSW who had history of care, were on ART, and had suppressed viral load and we used Poisson regression to estimate the associations of demographic characteristics and transmission risk behaviors with each outcome.

**Results:**

HIV seroprevalence was 69% (n = 138). Among all FSW the median age was 24 years (IQR: 22–28). Among the 20% who were newly diagnosed and reported previously testing negative, the median time since last HIV test was 11 months (interquartile range: 3–17). The majority (69%) of HIV-infected FSW had a history of HIV care, 52% reported current ART use, and 45% were virally suppressed. Of the FSW who reported current ART use, 86% were virally suppressed. Transmission risk behaviors were not associated with continuum outcomes.

**Conclusions:**

FSW in Lilongwe were predominately young and have a high HIV prevalence. Only half of HIV-infected FSW reported current ART use, but the majority of those on ART were virally suppressed. To reduce ongoing transmission and improve health outcomes, increased HIV testing, care engagement, and ART coverage is urgently needed among FSW. Universal testing and treatment strategies for all FSW in Malawi must be strongly considered.

## Introduction

HIV prevalence among female sex workers (FSW) remains disproportionately high despite decades of prevention activities.[1–5] Globally, the HIV prevalence among FSW is 12%, with a higher prevalence of 37% in sub-Saharan Africa. HIV-infected FSW have a higher number of sexual partners compared to other women of reproductive age, increasing the likelihood of HIV acquisition, as well as transmission to their clients.[1, 6] Effective interventions are clearly needed to reduce HIV acquisition and transmission among this key population.

Anti-retroviral therapy (ART) sharply reduces HIV morbidity, mortality, and transmission.[7, 8] To experience these benefits of ART, HIV-infected persons must be diagnosed and receive HIV care and treatment. The HIV care continuum is a commonly used framework that provides cross-sectional descriptions of population-level engagement in HIV testing, care, and treatment.[9, 10] If HIV-infected FSW fail to attain optimal outcomes along this “HIV continuum,” they will not receive the clinical benefits of HIV care and treatment, and also will likely continue to transmit HIV.[11, 12]

The HIV care continuum has not been well characterized among FSW.[12–14] Estimates of ART initiation, adherence, and treatment outcomes among this key population in sub-Saharan Africa have been derived predominately from small, intensively followed cohorts within clinical trials whose experiences may not be representative of FSW more generally.[13] Previous estimates among samples of FSW recruited within the community have found large drop-offs along the HIV care continuum.[15–22] Among FSW in sub-Saharan Africa, HIV infection awareness and ART initiation is sub-optimal.[17, 18, 22, 23] Without estimates of engagement in HIV care and treatment among FSW outside of these intensively followed cohorts, the unmet needs of this population are difficult to assess within high HIV prevalence settings, such as Malawi.[24]

We conducted the first study to characterize the HIV care continuum among FSW in Lilongwe, Malawi, where the FSW HIV prevalence (70%) is one of the highest globally.[1] In addition to quantifying the continuum, we examined the associations between continuum outcomes and both demographic characteristics and transmission risk behaviors.

## Methods

### Study procedures

This study was designed and implemented through a collaboration among The University of North Carolina at Chapel Hill, UNC Project Malawi, and Theatre for a Change (TfaC), a non-governmental organization in Malawi with far-reaching relationships with local sex work stakeholders including FSW, local chiefs or (non-FSW) community leaders, police, and government ministries. The Malawian law does not explicitly criminalize sex work; however, “the keeping of brothels” and “living on the earnings of prostitution or influencing others to engage in prostitution” is illegal.[25] Law enforcers often perform night raids to arrest persons loitering in entertainment and public places, and FSW are most commonly arrested.[25] To navigate the potential legal implications of working with FSW, community engagement activities were undertaken within the well-established infrastructures of UNC Project-Malawi and TfaC.

This study is a descriptive, cross-sectional biological and behavioral evaluation among FSW in Lilongwe, Malawi. FSW were systematically recruited using venue-based sampling at venues where FSW are known to solicit sex.[26, 27] Venue-based sampling has documented success with identification of hidden populations, including FSW.[10, 26, 28–30] In 2011, the Family Planning Association of Malawi conducted a nationally representative survey among FSW. This study found nearly 40% were between 20–24 years of age, approximately 60% attended primary school, and 98% were born in Malawi, comparable to our sample of FSW.[25] Our outreach team comprised HIV testing counselors, interviewers, a study nurse, a male driver for the mobile clinic, and a peer FSW to facilitate approaching women at the venues.

From July through September 2014, FSW were recruited from 23 different venues within Lilongwe; primarily venues were bars and bottle shops. Thirteen venues were bars, six were guesthouses or lodges, two were bottle shops, and two were bottle shops and guesthouses. Women were approached by a member of our outreach team and asked to participate in a study for women at risk for HIV. We used the 2011 Family Planning Association of Malawi’s definition of sex work: someone “who had received money in exchange for sex either regularly or occasionally up to 12 months” prior to the survey.[25] Women were eligible for enrollment if they were ≥18 years of age and self-reported as a FSW. We identified a sample size of 200 to provide a balance of feasibility and timeliness of data collection. A total of 201 women were approached or inquired about participation. Of these, 1 was considered ineligible to participate due to being <18 years of age and 200 were eligible and agreed to participate, resulting in our final sample.

Trained field workers administered a structured behavioral survey for all consented FSW to obtain demographics, pregnancy history, transmission risk behaviors, HIV testing history, and engagement in HIV care and treatment. The survey was translated from English to Chichewa, the predominant language in Malawi, and back translated. The survey was available in both English and Chichewa.

HIV serostatus was confirmed by trained HIV testing counselors for all participating FSW using Malawian National HIV Testing and Counseling guidelines, which indicate serial HIV-antibody rapid tests, Determine HIV-1/2 and Uni-Gold rapid HIV-antibody. Pre- and post-HIV test counseling, clinic referral, and risk reduction counseling were administered, and both male and female condoms were offered. FSW were defined as HIV-seronegative with a negative Determine HIV-1/2 test result or HIV-seropositive with positive Determine HIV-1/2 and Uni-Gold test results.

For FSW with confirmed HIV infection, CD4 measurements and plasma HIV-1 RNA levels were obtained by trained HIV testing counselors or study nurse. CD4 measurement was obtained using the Pima CD4^™^ analyzer (Alere PIMA CD4, Waltham, MA, USA), a self-contained, cartridge-based test.[31, 32] The results were provided to the participant within approximately 20 minutes and a trained study nurse was available to assist with interpretation of these results.

Dried blood spots (DBS) were collected to determine HIV-1 RNA concentrations.[33–35] Samples were stored in drying boxing with a humidity indicator card to monitor moisture levels, until they were brought to the UNC Project laboratory. HIV-1 RNA levels were available within 4 weeks of DBS collection and results were provided by a trained study nurse.

All FSW were referred to Lighthouse Trust HIV Clinic in Lilongwe, or nearest clinic, for follow-up examinations, HIV testing, care, and treatment as needed. The Lighthouse Trust HIV Clinic provides HIV testing and counseling, HIV primary care, management of opportunistic infections, ART care, cervical cancer screening, and family planning for all people living with HIV, including FSW. Clinics were chosen based on their experience for providing FSW-friendly care.

### Continuum outcome classifications

To assess whether women testing positive were newly (vs. previously) diagnosed, FSW were asked to report the date and results of their most recent HIV test. FSW who were seropositive and self-reported being HIV-negative at their most recent HIV test (or never testing previously) were defined as a new HIV diagnosis; those reporting being HIV-positive at their most recent test were classified as previously diagnosed.[36, 37] Previously diagnosed FSW who responded yes to the question “Have you ever seen an HIV health care provider?” or reported current ART use were classified as having history of HIV care.[36] Previously diagnosed FSW who answered yes to the question “Are you currently on ART?” were classified as being on ART. ART-eligible FSW were defined as those reporting current ART use, a CD4 ≤500 cells/mm^3^ following the Malawi national guidelines, currently pregnant or breastfeeding, or any pregnancy after July 2011 following adoption of Option B+ policy.[38] Option B+ provides confirmed HIV-infected pregnant and breastfeeding women lifelong ART regardless of CD4 count or clinical stage. ART adherence was assessed based on responses to the question “In the last 4 weeks, when was the last time you missed taking any of your anti-HIV medications?” (within the past week; 1–2 weeks ago; 2–4 weeks ago; or never skipped medications in the last 4 weeks)—adapted from the AIDS Clinical Trials Group measures for ART adherence for shorter recall time.[39] FSW were defined as virally suppressed with an HIV-1 RNA ≤5000 copies/mL and defined as undetectable with an HIV-RNA ≤550 copies/mL, the World Health Organization’s (WHO) recommended thresholds when using fingerstick DBS, which have good clinical compliance when compared to plasma.[40–42] DBS viral load thresholds are higher than plasma viral suppression thresholds due to the identification of cell-associated virus. Nonetheless, these thresholds are comparable.[41, 42]

### Data analyses

We used frequency distributions and descriptive statistics to characterize the study population. Proportions and associated 95% confidence intervals (CI) were computed for HIV serostatus and three key continuum outcomes: history of HIV care, current ART use, and viral suppression. We characterized these continuum indicators using two different approaches. The first approach, which provides information about opportunities for HIV transmission from FSW to their sexual partners, is estimated using all HIV-infected FSW, including those newly diagnosed and those previously diagnosed, as the denominator. For example, the portion of FSW who have a history of care has the denominator of FSW who report previously being diagnosed and those who are newly diagnosed through our study. The second approach, which more directly highlights healthcare delivery gaps for HIV prevention and treatment services, is estimated using the number of HIV-infected FSW achieving the prior step in the continuum. For example, under the second approach, the proportion of FSW who have a history of care has the denominator of FSW who reported being previously diagnosed. We report separately the small proportion of apparent discrepancies across the three key continuum outcomes. In addition to these analyses of the continuum outcomes, we also assessed ART use in relation to ART eligibility, and ART adherence among those on ART.

Demographic characteristics and transmission risk behaviors associated with HIV care continuum outcomes were explored.[43–46] A parsimonious set of demographic and transmission risk behaviors, which was established based on literature review and sample size considerations, included: age (18–24, 25–29, ≥30 years),[47] years in sex work (<1, 1.0–1.9, 2.0–2.9, ≥3.0 years),[48] weekly number of clients (≤19, ≥20 clients),[49] and condom use during vaginal sex with clients in prior 7 days (consistent use, inconsistent use).[49] The question used to assess condom use was “How often did you use condoms during vaginal sex with a paying sexual client in the last 7 days?” (never, rarely, sometimes, most times, and always). Consistent use included “always” responses and all other responses were classified as inconsistent use. We assessed the association between these behaviors and the three key HIV care continuum outcomes: a) previous diagnosis (vs. new diagnosis) among all HIV-infected FSW, b) history of HIV care (vs. no history of care) among those previously diagnosed, c) reported current ART use (vs. no current ART use) among those previously diagnosed and ART-eligible. We examined associations using Poisson regression with robust variance estimates to estimate prevalence ratios (PR) with 95% CI.[50]

### Sensitivity analyses

We conducted two separate sensitivity analyses to address a small number of apparent discrepancies across key continuum indicators. First, FSW who were virally suppressed but self-reported not being previously diagnosed were re-assigned as seronegative to account for the possibility of false-positive HIV rapid tests. Second, FSW who were virally suppressed but whose self-reports suggested they had not been previously diagnosed or did not report current ART use were re-assigned to categories in which they were assumed to be previously diagnosed, have a history of care, and be current ART users. Following re-classification of these FSW, we re-calculated the HIV seroprevalence and key continuum outcomes.

All statistical analyses were conducted using SAS software version 9.3 (SAS Institute, Cary, NC, USA).

### Ethical approval

The research protocol, survey, and consent forms were reviewed and approved by the Non-Biomedical Institutional Review Board at the University of North Carolina and the Malawi Ministry of Health and Population National Health Sciences Research Committee. All participants provided written informed consent. All study related activities were conducted in a safe and private location at the recruitment venue.

## Results

Among the total study population (n = 200), the median age was 24 years (IQR: 22–28). Most (66%) had not completed primary school and the majority (81%) were separated from their husband, divorced, or widowed (Table 1). Approximately 60% reported currently living in a bar or bottle shop, and 90% solicited clients at these venues. The median duration of exchanging sex for money was 3 years (IQR: 1–5). The median reported number of clients in the past 7 days was 21 (IQR: 10–35). Nearly three quarters reported consistent condom use during vaginal sex with clients in the past 7 days.

**Table 1:** Sociodemographics and sex work characteristics of female sex workers in Lilongwe, Malawi, July-September 2014.

	Total Population (n = 200)		HIV seronegative (n = 62)		HIV seropositive (n = 138)	
	n	(%)	n	(%)	n	(%)
Age (years)						
18–24	101	(51)	44	(71)	57	(41)
25–29	54	(27)	10	(16)	44	(32)
≥30	45	(22)	8	(13)	37	(27)
Nationality						
Malawian	195	(98)	62	(100)	133	(96)
Other	5	(2)	0	(0)	5	(4)
Education						
Never attended school	15	(7)	5	(8)	10	(7)
Some primary	117	(59)	36	(58)	81	(59)
Completed primary	20	(10)	3	(5)	17	(12)
Some secondary	44	(22)	15	(24)	29	(21)
Completed secondary	4	(2)	3	(5)	1	(1)
Marital status*						
Never married	28	(14)	13	(21)	15	(11)
Married (legal or traditional) or co-habitating	9	(4)	3	(5)	6	(4)
Separated, divorced, or widowed	162	(81)	45	(73)	117	(85)
Housing						
Private house	27	(13)	10	(16)	17	(12)
Bar or Bottle shop	115	(58)	35	(57)	80	(58)
Guesthouse or hotel	58	(29)	17	(27)	41	(30)
Number of pregnancies						
0	15	(7)	4	(6)	11	(8)
1	56	(28)	19	(31)	37	(27)
≥2	129	(65)	39	(63)	90	(65)
Duration of sex work (years)*						
<1.0	25	(12)	10	(16)	15	(11)
1.0–1.9	39	(20)	15	(24)	24	(17)
2.0–2.9	34	(17)	12	(19)	22	(16)
≥3.0	100	(50)	23	(37)	77	(56)
Location for soliciting clients						
Bar or bottle shop	181	(91)	57	(92)	124	(90)
Other	19	(9)	5	(8)	14	(10)
Number of clients per week*						
<10	43	(21)	16	(27)	27	(20)
10–19	45	(23)	12	(19)	33	(24)
20–29	52	(26)	19	(31)	33	(24)
≥30	58	(29)	14	(23)	44	(32)
Condom use with clients in past 7 days**						
Inconsistent	49	(24)	13	(21)	36	(26)
Consistent	151	(76)	49	(79)	102	(74)

*Missing data due to not knowing or refused to answer: marital status: n = 1; number of years exchanging sex for money: n = 2; number of clients in past 7 days: n = 2

**Inconsistent includes FSW who responded “never”, “rarely”, “sometimes”, or “most times”; Consistent includes FSW who responded “always”.

### HIV diagnosis

The HIV seroprevalence was very high (69%, 95% CI: 62%, 75%, n = 138). Among the HIV-infected FSW, 20% (95% CI: 13%, 26%, n = 27) were newly diagnosed (Table 2); of these, 74% (95% CI: 54%, 89%, n = 20) had tested negative previously, 19% (95% CI: 6%, 38%, n = 5) had never tested, and 7% (95% CI: 1%, 24%, n = 2) had tested but not received results. Among newly diagnosed FSW that had previously tested negative, the median time since last HIV test was 11 months (IQR: 3–17). The median CD4 among all newly diagnosed FSW was 464 cells/mm^3^ (IQR: 276–632) and median viral load was 44, 846 copies/ml (IQR: 5,981–202,395). Among previously diagnosed HIV-infected FSW, median time since diagnosis was 14 months (IQR: 4–44). The median CD4 was 528 cells/mm^3^ (IQR: 355–747) and the median viral load was 0 copies/ml (IQR: 0–31,295).

**Table 2:** HIV testing history, CD4 count, and VL at the time of cross-sectional survey by new and previous HIV diagnosis for female sex workers, (n = 138).

	New HIV diagnosis (n = 27)	Previous HIV diagnosis (n = 111)
	Median (Interquartile Range)	Median (Interquartile Range)
Time since last HIV test (months)*	11 (3–17)	14 (4–44)
CD4 count, cells/mm^3^	464 (276–632)	528 (355–747)
Viral load, copies/ml	44, 846 (5,981–202,395)	0 (0–31,295)

*Among those who report previously testing negative among new HIV diagnoses

### History of HIV care

**Fig 1:**
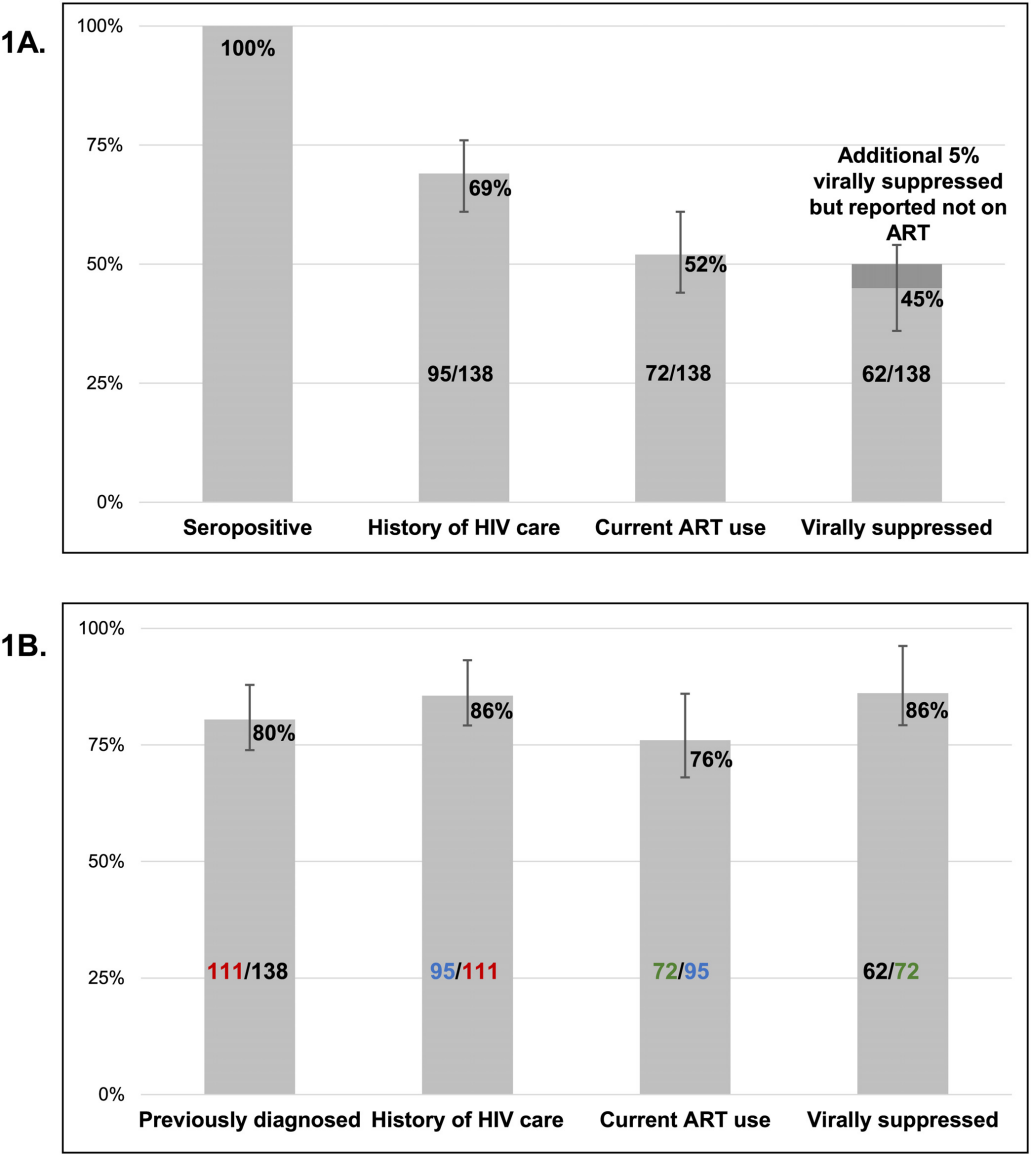
HIV Care Continuum among HIV-infected female sex workers, Lilongwe, Malawi (n = 138). 1A) Among all HIV-infected FSW; 1B) Among FSW achieving prior step. For Fig 1A, 69% (95/138) of all HIV-infected FSW had a history of care. Fifty-two percent (72/138) of all HIV-infected FSW reported current ART use and 45% (62/138) of all HIV-infected FSW were virally suppressed. For Fig 1B, 80% (111/138) of all HIV-infected FSW were previously diagnosed. While 86% (95/111) of FSW previously diagnosed had a history of HIV care. Seventy-six percent (72/95) of FSW with a history of HIV care reported current ART use. Eighty-six percent (62/72) of FSW reporting current ART use were virally suppressed.

Among all HIV-infected FSW, 69% (95% CI: 61%, 76%, n = 95) had a history of HIV care, as determined by report of ever seeing an HIV care provider or current ART use (Fig 1A). Among FSW previously diagnosed, 86% (95% CI: 78%, 92%, n = 95) had a history of HIV care (Fig 1B).

### Current ART use

Among the 52% (95% CI: 44%, 61%, n = 72) of all HIV-infected FSW who were currently on ART. (Fig 1A). Three- fourths (76%, 95% CI: 66%, 84%, n = 72) of previously-diagnosed FSW with a history of care reported current ART use (Fig 1B). Among all those reporting current ART use, adherence was suboptimal: 33% (95% CI: 23%, 45%) reported skipping pills within the past 4 weeks. Of those skipping pills, 52% (95% CI: 31%, 73%) reported skipping in past weeks, 17% (95% CI: 5%, 39%) in the past 1–2 weeks, 30% (95% CI: 13%, 53%) in the past 3–4 weeks.

About half of all HIV-infected FSW (48%; 95% CI: 35%, 56%, n = 66) reported no current ART use; their median CD4 was 477 cells/mm^3^ (IQR: 321–656). Of those that reported no current ART use, 62% (95% CI: 49%, 74%, n = 41) were eligible for ART; 41% (95% CI: 26%, 58%, n = 17) were newly diagnosed and 59% (95% CI: 42%, 74%, n = 24) were previously diagnosed. Among the previously diagnosed with no current ART use and ART eligible (n = 24), 92% (95% CI: 73%, 99%, n = 22) had a CD4 ≤500 cells/mm^3^ and 8% (95% CI: 1%, 27%, n = 2) were ART-eligible under Option B+ (currently pregnant, currently breastfeeding, or any pregnancy after July 2011).

### Viral suppression

Approximately half (45%, 95% CI: 36%, 54%, n = 62) (Fig 1A) of all HIV-infected FSW were virally suppressed (≤5000 copies/mL) and 38% had an undetectable viral load (≤550 copies/mL). Among previously diagnosed FSW (n = 111), half had undetectable viral loads (median viral load = 0 copies/ml IQR: 0–31,295) (Table 2). Of the FSW who reported current ART use, 86% (95% CI: 76%, 93%, n = 62) had viral loads ≤5000 copies/mL (Fig 1B). Seven women (5%) of all HIV-infected FSW were identified as virally suppressed but did not report current ART use or previous HIV diagnosis.

### Sensitivity analyses

Among the seven FSW who were virally suppressed but did not report experience with “upstream” continuum indicators, four reported previous diagnosis and a history of care but no current ART use, and three had no indication of previous diagnosis, history of care, or current ART use based on self-report.

In the first sensitivity analysis that re-classified the three FSW who self-reported not being previously diagnosed as seronegative, the HIV seroprevalence and HIV care continuum outcomes were similar to those of the main analyses. Of all the 200 FSW enrolled in our study, 68% were seropositive (95% CI: 61%, 74%). Among HIV-infected FSW, 70% (95% CI: 62%, 78%) had a history of care, 53% (95% CI: 45%, 62%) reported current ART use, and 46% (95% CI: 37%, 55%) were virally suppressed.

In the second sensitivity analysis that re-classified these seven FSW as being previously diagnosed, with a history of care and current ART use, the overall proportions achieving the HIV care continuum outcomes were similar to those obtained in the main analyses. Of all HIV-infected FSW, 72% (95% CI: 64%, 80%) had a history of care, 57% (95% CI: 49%, 66%) reported current ART use, and 50% (95% CI: 41%, 59%) were virally suppressed. Among previously diagnosed FSW (83%, (95% CI: 75%, 89%), 88% (95% CI: 80, 93%) had a history of care. Of those with a history of care, 79% (95% CI: 70%, 87%) FSW currently ART using and among those with current ART use, 87% (95% CI: 78%, 94%) were virally suppressed.

### Factors associated with HIV care continuum outcomes

In exploratory multivariable analyses (Table 3), there was little to no association between age, duration in sex work, number of weekly clients and condom use and the key HIV care continuum outcomes: previously diagnosed, history of HIV care, and current ART use.

**Table 3:** Associations of demographic and transmission risk behaviors with HIV care continuum outcomes (previously HIV diagnosed, history of HIV care, and current ART use) among HIV-infected female sex workers in Lilongwe, Malawi.

	Previously HIV diagnosed (n = 138)		History of HIV care (n = 111)		Current ART use (n = 96)	
Characteristic	PR (95% CI)	APR* (95% CI)	PR (95% CI)	APR* (95% CI)	PR (95% CI)	APR* (95% CI)
Age (years)						
18–24	0.89 (0.40, 2.01)	0.74 (0.30, 1.84)	0.91 (0.78, 1.07)	0.99 (0.86, 1.14)	0.90 (0.67, 1.17)	0.98 (0.74, 1.31)
25–29	0.84 (0.35, 2.02)	0.81 (0.34, 1.91)	0.87 (0.72, 1.04)	0.89 (0.73, 1.08)	0.88 (0.67, 1.17)	0.93 (0.70, 1.23)
≥30	1.00	1.00	1.00	1.00	1.00	1.00
Duration of sex work (years)						
<1.0	1.58 (0.60, 4.19)	1.81 (0.62, 5.33)	1.02 (0.83, 1.25)	1.02 (0.83, 1.12)	0.78 (0.49, 1.25)	0.81 (0.49, 1.33)
1.0–1.9	1.73 (0.78, 3.83)	2.04 (0.85, 4.94)	0.79 (0.58, 1.09)	0.84 (0.63, 1.12)	0.82 (0.56, 1.20)	0.91 (0.62, 1.33)
2.0–2.9	0.81 (0.25, 2.58)	0.91 (0.28, 2.88)	0.95 (0.76, 1.17)	0.93 (0.75, 1.15)	0.87 (0.62, 1.21)	0.87 (0.60, 1.24)
≥3.0	1.00	1.00	1.00	1.00	1.00	1.00
Number of clients per week						
≤19	0.64 (0.31, 1.33)	0.67 (0.33, 1.36)	1.13 (0.98, 1.31)	1.12 (0.96, 1.30)	1.11 (0.89, 1.39)	1.10 (0.88, 1.38)
≥20	1.00	1.00	1.00	1.00	1.00	1.00
Condom use**						
Inconsistent	1.42 (0.70, 2.86)	1.65 (0.79, 3.47)	0.94 (0.77, 1.14)	0.90 (0.75, 1.01)	1.12 (0.88, 1.43)	1.08 (0.84, 1.38)
Consistent	1.00	1.00	1.00	1.00	1.00	1.00

PR: Prevalence Ratio; APR: Adjusted Prevalence Ratio; CI: Confidence Interval

*Adjusted for all other variables in table

**Inconsistent includes FSW who responded “never”, “rarely”, “sometimes”, or “most times”; Consistent includes FSW who responded “always”.

## Discussion

In this study, FSW in Lilongwe, Malawi were predominantly young and heavily HIV burdened, with one-fifth of HIV-infected FSW being previously unaware of their HIV infection. Although most HIV-infected FSW reported seeing an HIV healthcare provider at least once, only half reported current ART use. More encouragingly, among FSW on ART, over four-fifths were virally suppressed, the ultimate goal for clinical and transmission outcomes. Both HIV-infected and HIV-uninfected FSW in our sample were engaging in high-risk sexual behaviors, including multiple partners per week and inconsistent condom use. Among HIV-infected FSW, transmission risk behaviors were not statistically associated with being previously diagnosed, having a history of HIV care, or current use of ART.

Despite continued HIV prevention efforts in Malawi and a steady decline in overall prevalence in this setting,[24] HIV seroprevalence among this sample of FSW in Lilongwe remains consistent with estimates among urban FSW in Malawi from nearly a decade ago.[30] The prevalence that we observed may be an overestimate for all FSW in Malawi, given that our participants were recruited from venues in the capital and second-largest city in Malawi. However, current national HIV prevalence estimates among FSW are unavailable for comparison. The cross-sectional nature of this study does not allow for ascertainment of the time or source of HIV infection. Although it is likely FSW acquired HIV through sex work,[3] another potential source of infection may have been from previous husbands given the the majority of FSW within our sample were separated or divorced. The consistently high HIV prevalence among urban FSW in Malawi over the past decade highlights the need for combination behavioral, biomedical, and structural HIV prevention and treatment efforts for both HIV-uninfected and infected FSW.

The proportion of FSW who were newly diagnosed in our study has serious consequences for both FSW and their sexual partners. Similar to FSW in South Africa and Zimbabwe,[18, 23] approximately 20% of FSW in our sample were unaware of their HIV infection. Establishing a timely HIV diagnosis for FSW is absolutely critical.[51] FSW who remain unaware of their HIV infection miss opportunities for entry into HIV care, leading to later ART initiation, higher risk for HIV clinical progression, and ongoing transmission.[52] Currently, the WHO recommends that high-risk persons, such as FSW, undergo HIV testing at least every 12 months.[53] FSW within our sample reported multiple clients per week and inconsistent condom use providing a high frequency of possible HIV acquisition and transmission. Frequent HIV testing strategies, such as testing every three to six months, should be explored within the context of current resources in Malawi. The time between tests and proportion unaware of their HIV infection identified in our study highlights the need for frequent outreach to encourage rapid HIV testing in this high-risk population.

It is also notable that the proportion of previously diagnosed FSW is higher than previous estimates among FSW in sub-Saharan Africa.[18, 25, 54] Given that nearly all FSW within in our sample reported previous pregnancies, it is likely that they received HIV testing and counseling through routine antenatal care. Furthermore, FSW in our sample could have participated in TfaC sexual and reproductive health promotion programs. Therefore, our sample of FSW may be more likely to be aware of their HIV infection.

Overall in this sample, about half of HIV-infected FSW were not on ART and therefore not receiving the full clinical, immunological, and transmission prevention benefits of therapy. The proportion of HIV-infected FSW not on ART in our sample was lower when compared to the approximately 62% to 75% of HIV-infected not on ART in Zimbabwe.[18] Despite the recognition that timely ART uptake can improve health outcomes and decrease onward transmission,[37, 55, 56] there are currently few interventions specifically focusing on increased access and adherence to ART for FSW in sub-Saharan Africa.[21, 55] However, the recent expansions of ART may increase ART initiation among FSW in Malawi. Since 2011, Malawi has provided lifelong ART for all women who are pregnant or breastfeeding for the prevention of mother-to-child transmission under Option B+.[57] The Option B+ program within Malawi has been highly successful in providing treatment for HIV-infected pregnant and breastfeeding women.[58, 59] We noted nearly all FSW in our sample reporting a previous pregnancy, suggesting that antenatal could be an opportune time for HIV-infected FSW not ART to initiation ART. In addition to Option B+, Malawi implemented earlier initiation of ART (CD4 count ≤500 cells/mm^3^) in April 2014.[60] Further expansion of ART initiation should be prioritized to reach all HIV-infected FSW in Malawi.

Within our exploratory analysis, we identified little to no association between transmission risk behaviors and key HIV care continuum outcomes. These results are in contrast to observed reductions in sexual risk behaviors following HIV diagnosis[43] and initiation of HIV care and treatment among non-FSW populations.[44–46] The null association between behaviors and continuum outcomes in our FSW population may be due to the nature of their work: because their livelihoods depend on high-risk sexual behaviors, FSW may face greater difficulty in reducing transmission risk behaviors following diagnosis and engagement in care than do other populations.[1]

The high HIV prevalence identified in our study also highlights the need for HIV prevention among HIV-uninfected FSW. Although it has not yet been evaluated specifically among FSW, pre-exposure prophylaxis has emerged as an effective biomedical intervention that could provide a highly appropriate prevention strategy for at risk FSW.[61] Given the multilevel HIV risks FSW face, PrEP must be complemented with other proven effective interventions, such as community empowerment and testing and treatment strategies to reduce HIV incidence in FSW and their clients.[62] Comprehensive prevention packages that incorporate PrEP delivery must be developed and rigorously evaluated among FSW.

In this study, the HIV care continuum provided a valuable framework for describing HIV testing, care, and treatment cross-sectionally among FSW in Lilongwe, Malawi; however, the framework fails to fully capture the dynamic nature of FSW’s engagement in the trade and with HIV prevention and care services. For example, the traditional continuum framework does not explicitly describe important processes, such as care re-engagement and viral re-suppression that may occur for some HIV-infected FSW.[13] A related limitation is that we assessed HIV care history but not current HIV care status; therefore, we were unable to determine whether suboptimal ART use among ART-eligible FSW was due to care dis-engagement or rather failures in ART initiation/re-initiation among FSW who were currently in care. Prospective, longitudinal studies that can better enumerate dynamic, multidirectional movement would provide additional, useful understanding of HIV testing, care, and treatment of FSW.

The outcomes we used to describe the HIV care continuum were mostly reliant on self-report and thus subject to misclassification. In particular, our estimated proportion of newly diagnosed FSW may be an overestimate of new diagnoses if women were uncomfortable reporting their known HIV-positive status. Additionally, FSW who were previously diagnosed but not on ART may have felt it more socially desirable to report not being previously diagnosed and therefore unable to be on ART. Furthermore, FSW who were virally suppressed but did not report engaging in “upstream” care continuum outcomes may have been concerned about reporting HIV status awareness while continuing to be engaged in sex work. This apparent discrepancy related to virally suppressed FSW that are reportedly unengaged in HIV care and not on ART, which may be partially explained by the possibility that some small percentage were elite controllers,[63] has been seen previously among FSW in Zimbabwe.[18] Clinical records could potentially have provided more reliable diagnosis and ART use data; however, such data are not readily available in Malawi.

This study was the first to characterize key HIV care continuum outcomes among a sample of FSW in Lilongwe, Malawi—where the HIV prevalence among FSW remains among the highest globally. HIV-burdened FSW in sub-Saharan Africa are severely understudied and underserved, leading to limited understanding of and attention to their HIV testing, care, and treatment engagement. Our study suggests an enormous need for integrated behavioral, biomedical, and structural approaches for both HIV-infected and HIV-uninfected FSW to improve clinical outcomes and prevent onward transmission.
